# Editorial: Computational genomic and precision medicine

**DOI:** 10.3389/fgene.2025.1631668

**Published:** 2025-05-29

**Authors:** Zeeshan Ahmed, Ramkumar Thirunavukarasu, Atlas Khan

**Affiliations:** ^1^ Department of Medicine, Division of Cardiovascular Diseases and Hypertension, Robert Wood Johnson Medical School, New Brunswick, NJ, United States; ^2^ Institute for Health, Healthcare Policy and Aging Research, Rutgers Health, New Brunswick, NJ, United States; ^3^ School of Computer Science, Engineering and Information Systems, Vellore Institute of Technology University, Vellore, India; ^4^ Department of Medicine, Columbia University Irving Medical Center, Columbia University, New York, NY, United States

**Keywords:** computational genomics, precision medicine, artificial intelligence, machine learning, deep learning, multi-omics

## 1 Introduction

Misdiagnosis has been reported among the leading causes of death, along with cancer, heart disease, and respiratory diseases (Toker et al., 2024). A fair amount of literature has been published in impactful peer-reviewed journals, which discuss medication error and delayed treatment, and is accessible through authentic resources (e.g., PubMed) (Ahmed et al., 2020). One of the most trending subjects in life sciences, which addresses these issues and contributes to providing personalized treatment to patients, is genomic and precision medicine (Ahmed, 2020). It involves patient engagement, analyzing medical records to examine provided diagnoses and treatment outcomes, and investigating the genomic profile to understand disease mechanisms and propose better treatments (Ahmed, 2022). Furthermore, it promotes integrating and analyzing different kinds of patient data (e.g., clinical, sociodemographic, behavioral, biomedical image, and multi-omics) to form multimodal data to discover important risk factors and biomarkers, which could be used to prevent and predict diseases (Singh et al., 2025). This Research Topic focuses on gathering the most up-to-date knowledge on recent advances in analytical approaches, including deep and machine learning models for identifying disease-associated genes and rare variants, and predicting the best treatment outcomes for genomic and precision medicine. Successfully achieving the goals of this Research Topic, we were able to publish five interesting peer-reviewed articles.

In, “*SAFE-MIL: a statistically interpretable framework for screening potential targeted therapy patients based on risk estimation*”, Guan et al. set out to construct a generalizable framework for risk assessment of treatment failure for Non-Small Cell Lung Cancer (NSCLC) patients receiving epidermal growth factor receptor tyrosine kinase inhibitor-based treatment. Currently, patients with NSCLC who have the same target gene mutation experience vastly different treatment outcomes, largely due to varying mutation abundance levels and drug sensitivity that existing models don’t account for, leading to black boxes and misalignment with Food and Drug Administration (FDA) standards, weakening the clinical applicability of machine learning (ML)-driven drug prediction models. This study utilized three independent patient cohorts, implementing clinical and genomic data (SNVs, indels, mutation abundance levels) to create drug effectiveness labels. Unsupervised k-means clinically similar patient clustering and a multi-instance learning model were used, incorporating a custom Hosmer-Lemeshow-based test for loss function. SAFE-MIL predicted risk scores at a lower prediction error compared to baseline models, along with identifying a mutation abundance threshold (0.479), stratifying patients into risk categories. This model excels in assessing treatment for many patients facing stratification problems in the clinical context.

In, “*Multi-fusion strategy network-guided cancer subtypes discovering based on multi-omics data*”, Liu et al. aimed to develop Self-supervised Multi-fusion Strategy Network (SMMSN): a dual, self-supervised, multi-omics multi-fusion-based model that identifies cancer subtypes. Past methods excel in feature-level representation but are lacking in patient-patient molecular profile similarity analysis, and this absence of structural insights limits clinical precision. Here, Stacked Autoencoder Network (SAE) was employed to learn expression patterns for each omics type (mRNA, DNA methylation, miRNA) while Graph Convolutional Networks (GCN) were used to learn structure-based representations from K-nearest neighbor (KNN) networks, followed by multi-omics fusion incorporating error reconstruction and adaptive weighting, which funneled into dual self-supervised learning to generate clustering probability distributions. This study utilized 8 independent datasets, with labeled data from kidney cancer, Alzheimer’s, and low-grade glioma, while unlabeled datasets included glioblastoma, breast, kidney, lung, and colon cancer. Clustering accuracy was ultimately higher for SMMSN than for any other comparable multi-view clustering algorithm or deep learning (DL)-based method, as tested on the three labeled datasets (SMMSN scoring ACCs of 85.34, 68.83, and 65.80, respectively). SMMSN’s methodology applies to many cancers, enhancing tailored treatment and predictive prognosis.

In, “*MSFN: a multi-omics stacked fusion network for breast cancer survival prediction*”, Zhang et al. incorporated a novel Multi-omics Stacked Fusion Network (MSFN) methodology to predict breast cancer survival risk in a cohort of 1,048 patients. Breast cancer has become the most prevalent cancer in the world, and survival risk is an important step in treatment recommendations, but previous single-omics-reliant methods are limited in their accuracy potential, and current DL methods are incompatible with multi-omics. MSFN constructed patient similarity networks using similarity network fusion to connect similarity between patients with correlation of multi-omics data (gene expression and copy number variation data), then constructed a Residual Graph Convolutional Network (ResGCN) to extract prognostic information, further feeding results into AdaboostRF for survival prediction. Ten-fold cross-validation results demonstrated the accuracy of MSFN (AUC of 0.9787 and accuracy of 0.991) as compared to previous methods, and when excluding native features from MSFN, further succeeding in different survival cohorts. MSFN is therefore superior in both short and long-term survival prediction, aiding clinical decision-making.

In, “*Integrative multi-omics summary-based mendelian randomization identified key oxidative stress-related genes as therapeutic targets for atrial fibrillation and flutter*”, Chen et al. integrated a summary statistics-based approach on multi-omics data for a more comprehensive understanding of the connection between Oxidative Stress (OS) and Atrial fibrillation (AF). Currently, it is known that OS is implicated in the pathogenesis of AF, but knowledge is limited in the exact contributions of OS and the causal interaction with AF. GWAS was integrated with multiple SNP-based Quantitative Trait Loci (QTL) studies (methylation, gene, and protein-based). Summary-based Mendelian Randomization (SMR) analyzed if SNPs’ association to omics traits had an impact on AF risk, employing the HEIDI test to eliminate linkage and pleiotropy as confounders, while also incorporating Bayesian co-localization to confirm shared causal variants. Importantly, the *TTN* gene was found to play a protective role in AF, and methylation at two CpG sites was associated with increased *TTN* expression and thereby lower AF risk; *ALAD* and *APOH* were important proteins associated with a lower risk of AF. The SMR approach proves valuable in elucidating the contributions of OS-related genes in the landscape of AF while eliminating potential confounders, leading to novel causal relationships.

In, “*Prognostic value of four immune-related genes in lower-grade gliomas: a biomarker discovery study*”, Wang et al. aimed to investigate the relationship between multiple immune-related genes (IGG) and low-grade glioma (LGG), leveraging past methods that mainly focused on single gene relationships to LGG. Although there have been significant advancements in the treatment of LGGs, they tend to recur and develop drug resistance, necessitating the discovery of innovative biomarkers that elucidate precise pathological mechanisms. IGGs obtained from the ImmPort database were intersected with the DEG profile between RNA-seq-based control samples and the glioblastoma patient samples, yielding statistically significant DEGs, validated experimentally by qRT-PCR on case vs. control cell lines. Univariate Cox regression analysis and LASSO were employed to identify the most prognostic genes in their contribution to the survival of LGG patients, validated using an external cohort. Conclusively, a 4-gene prognostic model was constructed using *KLRC3, MR1, PDIA2*, and *RFXA*, further developing a nomogram based on these biomarkers to predict survival rates, demonstrating great potential for clinical uptake in the assessment of survival, risk stratification, and tailored treatments.
